# Latest progress and challenges in drug development for degenerative motor neuron diseases

**DOI:** 10.4103/NRR.NRR-D-24-01266

**Published:** 2025-05-06

**Authors:** Xiangjin Wen, Tianxiang Lan, Weiming Su, Bei Cao, Yi Wang, Yongping Chen

**Affiliations:** 1 Department of Neurology, West China Hospital, Sichuan University, Chengdu, Sichuan Province, China; 2 West China School of Medicine, West China Hospital, Sichuan University, Chengdu, Sichuan Province, China; 3 Department of Pathophysiology, West China College of Basic Medical Sciences & Forensic Medicine, Sichuan University, Chengdu, Sichuan Province, China

**Keywords:** amyotrophic lateral sclerosis, clinical trial, degenerative motor neuron diseases, disease modifying therapy, drug development, motor neuron disease

## Abstract

Motor neuron diseases are sporadic or inherited fatal neurodegenerative conditions. They selectively affect the upper and/or lower motor neurons in the brain and spinal cord and feature a slow onset and a subacute course contingent upon the site of damage. The main types include amyotrophic lateral sclerosis, progressive muscular atrophy, primary lateral sclerosis, and progressive bulbar palsy, the pathological processes of which are largely identical, with the main disparity lying in the location of the lesions. Amyotrophic lateral sclerosis is the representative condition in this group of diseases, while other types are its variants. Hence, this article mainly focuses on the advancements and challenges in drug research for amyotrophic lateral sclerosis but also briefly addresses several other important degenerative motor neuron diseases. Although the precise pathogenesis remains elusive, recent advancements have shed light on various theories, including gene mutation, excitatory amino acid toxicity, autoimmunology, and neurotrophic factors. The US Food and Drug Administration has approved four drugs for use in delaying the progression of amyotrophic lateral sclerosis: riluzole, edaravone, AMX0035, and tofersen, with the latter being the most recent to receive approval. However, following several phase III trials that failed to yield favorable outcomes, AMX0035 has been voluntarily withdrawn from both the US and Canadian markets. This article presents a comprehensive summary of drug trials primarily completed between January 1, 2023, and June 30, 2024, based on data sourced from clinicaltrials.gov. Among these trials, five are currently in phase I, seventeen are in phase II, and eleven are undergoing phase III evaluation. Notably, 24 clinical trials are now investigating potential disease-modifying therapy drugs, accounting for the majority of the drugs included in this review. Some promising drugs being investigated in preclinical studies, such as ATH-1105, are included in our analysis, and another review in frontiers in gene therapy and immunotherapy has demonstrated their therapeutic potential for motor neuron diseases. This article was written to be an overview of research trends and treatment prospects related to motor neuron disease drugs, with the aim of highlighting the latest potentialities for clinical therapy.

## Introduction

Motor neuron diseases (MNDs) are a group of progressive neurological disorders that selectively affect the upper and/or lower motor neurons in the brain and spinal cord. The main types include progressive muscular atrophy (PMA), primary lateral sclerosis (PLS), progressive bulbar palsy (PBP), and amyotrophic lateral sclerosis (ALS), with disparities in their pathogenesis lying in the location of the lesions (Bjelica and Petri, 2024). The lesions of PMA predominantly occur in lower motor neurons (LMN), whereas those of PLS are located in the upper motor neurons (UMN). PBP is mainly characterized by the degeneration of a nucleus in the medulla oblongata motor nerve. Typically, ALS is a degenerative MND featuring the involvement of both UMN and LMN (Foster and Salajegheh, 2019). As one of the most prevalent and destructive forms of MND, ALS is highly heterogeneous, and survival rates fluctuate widely, with a median rate of about 3–5 years after symptom onset, but only approximately 10% of ALS patients surviving for more than 10 years (Tsekrekou et al., 2024). Globally, the prevalence of ALS amounts to approximately 4 to 7 cases per 100,000 head of population, and the annual incidence is around 1.59 new cases per 100,000 (Mehta et al., 2022; Izenberg, 2023). Patients typically exhibit muscle wasting, fasciculation, and in severe cases, difficulties in swallowing and even respiratory failure (Bonafede and Mariotti, 2017; Johnson et al., 2022; Vidovic et al., 2023). The etiology and risk factors influencing ALS’s progress remains unclear but may involve genetic mutations (Su et al., 2022) and exposure to environmental toxins (Su et al., 2021). Currently, more than 30 genes associated with ALS have been identified, including the Cu/Zn superoxide dismutase 1 gene (*SOD1*), chromosome 9 open reading frame 72 gene (*C9orf72*), the gene coding TAR DNA binding protein 43 (TARDBP/TDP43), and fused in sarcoma (*FUS*) (Renton et al., 2014; Zufiría et al., 2016; Mejzini et al., 2019; Shatunov and Al-Chalabi, 2021; Chen et al., 2022). Research has shown that the pathogenesis of ALS is likely to be associated with oxidative stress, mitochondrial dysfunction, aberrant protein accumulation, inflammation, and neural dysregulation (Ling et al., 2013; Islam, 2017; Jin et al., 2020). Although there are currently no drugs that can cure ALS, based on identified and validated pathogenesis mechanisms, a multitude of therapeutic drugs are being developed with potential efficacy in ameliorating patients’ symptoms and delaying disease progression.

In recent years, the US Food and Drug Administration (FDA) has approved five drugs for the treatment of ALS: riluzole (including different forms, such as Rilutek, Tiglutik, and Exservan), edaravone (Jaiswal, 2019), AMX0035, dextromethorphan hydrobromide and quinidine sulfate and tofersen (Blair, 2023). Owing to the rarity, restricted survival time, and severe symptoms of ALS, along with the ineffective performance of existing drugs in end-stage patients, the pathogenesis of ALS is undergoing extensive research, and diverse drugs targeting different mechanisms are being developed and approved. Agents, including fasudil (Takata et al., 2013), ibudilast (López-Blanch et al., 2023), and trimetazidine dihydrochloride (Scaricamazza et al., 2022), along with several other drugs, are currently undergoing clinical trials. Some are new drugs, while others are modifications of old drugs (including changes in dosage forms and recombinations of different drugs). Based on data from clinicaltrials.gov, this article summarizes the eligible clinical trials conducted between January 1, 2023 and June 30, 2024. We gathered data from a total of 28 clinical trials that met our standards, while excluding one that was terminated and one that had not been updated. We included 24 disease-modifying therapy (DMT)-associated, one non-DMT drug, and three stem-cell-related trials. Of these 28 trials, five specifically TAR DNA-binding protein 43 (TDP-43) aggregates were particularly of interest due to the universality of the pathology. Furthermore, to explore a more comprehensive development pipeline of the therapies available, we also summarized the progress made on gene therapy and immunotherapy approaches, which are considered highly promising (Sever et al., 2022).

## Search Strategy

For this review, we primarily gathered data from clinical trials via clinicaltrials.gov. We utilized “ALS” and “treatment” as the key search terms and employed advanced search options, including “recruiting,” “not yet recruiting,” “active, not recruiting,” and “completed,” to determine study status. Subsequently, we restricted our selection to drugs in phases 1, 2, and 3 with a primary completion date from January 1, 2023, to June 30, 2024. In the initial screening, studies that failed to meet the prerequisites, including those pertaining to ALS biomarkers, and investigations that were halted but whose status remained unamended on the website, were excluded. Consequently, a total of 28 drug studies fulfilling the search requirements were acquired. For the approved drugs detailed within the relevant section, we initially compiled a list of medications via www.als.org, and subsequently verified their status on the official Drugs@FDA portal. In the section of preclinical drugs, we employed PubMed as the database for a literature search, using “preclinical trials” as a fundamental criterion, and “ALS” and “treatment” as the core keywords. We identified eight studies that conformed to our search criteria, four of which yielded significant findings and were incorporated into our analysis. The remaining four studies were omitted on account of either their negative outcomes or unrelated subjects. Most significantly, it is anticipated that disparities will have emerged in the search outcomes due to the temporal incongruity between the search execution and updates to the experimental data. Such disparities should not have prejudiced our analysis of pharmaceutical advancements and the associated challenges within this review, and this bias has also been highlighted in the Prospects section.

## Motor Neuron Disease Subtypes

### Progressive muscular atrophy

PMA is a condition that typically emerges in adulthood, and despite the identification of familial occurrences, it is predominantly sporadic in nature (Rowland, 2010). It is approximated that PMA constitutes 2.5%–11% of all adult-onset MNDs. Clinically, PMA is characterized by LMN degeneration, involving symptoms such as muscle weakness, atrophy, fasciculation, and the attenuation or absence of tendon reflexes (Izenberg, 2023). This pattern of weakness and atrophy typically commences in the distal limb muscles in an asymmetric manner before advancing over a period of several months to years (Liewluck and Saperstein, 2015). Nevertheless, a growing body of longitudinal research has revealed that only 15% of individuals with MND exhibit isolated LMN involvement (Riku et al., 2014). Traditionally, isolated anterior horn cell degeneration has been regarded as a definitive pathological marker of PMA. However, post-mortem examinations have not only identified ubiquitinated inclusions akin to those seen in ALS within LMN but also corticospinal degeneration in 50% to 85% of PMA patients (Ince et al., 2003). Concurrently, some patients initially diagnosed with PMA may progressively manifest clinical indicators of UMN damage as the disease advances, indicating that these patients are, in reality, afflicted with ALS accompanied by concurrent LMN degeneration, a diagnosis that evolves with the clinical presentation. In comparison to ALS, the majority of PMA patients are male, and the age of onset is typically later. The average survival duration of PMA is significantly prolonged, surpassing that of ALS by 6–7 years, with a median survival period of 48 months (Yedavalli et al., 2018). In essence, the therapeutic principles of PMA and ALS are congruent (Jackson et al., 2015); however, vigilance is warranted regarding the potential progression of isolated PMA to ALS. Whenever feasible, it is imperative to closely monitor patients’ symptoms, neuronal pathology, and alterations in brain imaging within the clinical setting. This meticulous approach should prevent misdiagnosis, overlooked diagnosis, and exclusion from clinical trials, thereby ensuring that patients do not miss the opportunity to receive suitable therapeutic interventions (Kim et al., 2009).

### Primary lateral sclerosis

PLS is a selective UMN disease of unknown etiology (Statland et al., 2015) that, unlike ALS, does not involve the LMN. This distinction enables a differentiation between progressive ALS and PLS to be made based on the involvement of LMN, which can be identified through electromyography (Turner et al., 2020). PLS typically manifests after the age of 50, often beginning in the lower limbs with symmetrical leg weakness and elevated muscle tone. Other symptoms might comprise dysarthria, dysphagia, and ataxia (Vacchiano et al., 2024). Currently, the diagnosis of PLS is mainly based on exclusion and demands prolonged observation for confirmation, which frequently delays timely treatment. Currently, the treatment of PLS is largely symptomatic, focusing on alleviating specific symptoms. For example, baclofen and benzodiazepines are frequently employed to handle spasticity. Medications approved for ALS have demonstrated no verified benefits in PLS patients (Statland et al., 2015). Owing to the lack of identified causative genetic mutations, gene therapy has not been explored for PLS as of yet. Further investigations into the etiology and pathogenesis of PLS are of paramount importance to identify potential therapeutic targets and enhance treatment options.

### Progressive bulbar palsy

PBP is classified by the International Classification of Diseases as a variant of ALS and mainly involves the degeneration of motor neurons in the glossopharyngeal, vagus, and hypoglossal nerves. Similar to PLS, its precise cause remains unknown (Cerero Lapiedra et al., 2002). Patients with PBP demonstrate progressively deteriorating dysarthria and dysphagia as a result of bulbar cranial nerve degeneration. Additional manifestations encompass facial muscle weakness and palatal fasciculations (Moini et al., 2024). The prognosis for PBP is dismal, with an average survival period of 2 years, and numerous patients succumbing to complications such as aspiration pneumonia in the advanced stages of the disease (Lewis and Spillane, 2019). Currently, there are no effective treatments for PBP. Therefore, improving patients’ quality of life and minimizing the occurrence of late-stage complications remain critical areas of focus for managing this condition.

## Pathogenesis

Here, we initially summarize possible pathogeneses of MNDs in order to comprehend how potential drugs could function. As a highly heterogeneous neurodegenerative disease, ALS manifests and progresses via various etiologies. The majority of cases involve complex or unidentified genetic variants, leading to challenges in understanding the specific pathological mechanisms of ALS due to the potential genetic impact on multiple cellular processes (Akçimen et al., 2023). Moreover, this heterogeneity impedes the translation of mechanistic research into effective treatments. However, recent advancements in sequencing and biochemical technologies have facilitated the continuous identification of ALS-related disease genes and the proposal of several common hypotheses regarding its pathogenesis (Mejzini et al., 2019).

### Amyotrophic lateral sclerosis associated genes

**Figure 1:**
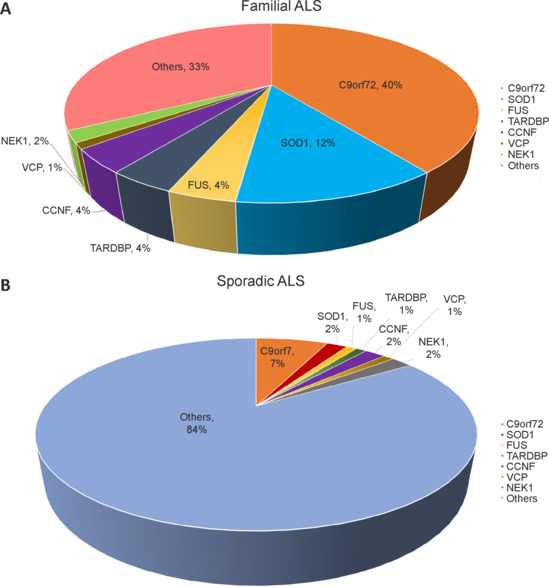
Statistical representation of the genes contributing to familial ALS (A) and sporadic ALS (B). The two graphical representations employing fan plots depict the sharp proportions of various pathogenic genes contributing to familial ALS compared with sporadic ALS. ALS: Amyotrophic lateral sclerosis; C9orf72: chromosome 9 open reading frame 72; CCNF: cyclin F; FUS: fused in sarcoma; NEK1: NIMA-related kinase 1; SOD1: superoxide dismutase 1; TARDBP: TAR DNA-binding protein; VCP: valosin-containing protein.

Among ALS cases, sporadic ALS (sALS) accounts for 80% to 90%, while familial ALS (fALS), inherited through autosomal dominant transmission, constitutes around 10% to 20%. Several identical pathogenic mutations have been identified in many cases of fALS (**Figure 1A** and **B**). Since the discovery of the first ALS-related gene mutation in *SOD1* three decades ago, the exploration of ALS-related mutated genes has been in full bloom, with *TARDBP*, *FUS*, *C9orf72*, *NEK1*, *CCNF*, and *VCP* among those identified (Elmansy et al., 2023). To this day, over 100 mutant genes have been discovered in individuals suffering from ALS.

#### Superoxide dismutase 1

A pathogenic variant of SOD1 was first identified in 1993, making it the first ALS-related gene to be discovered. SOD1 mutations account for 20% of fALS and 1%–2% of sALS incidences and are key genetic factors in Asian populations (Wang et al., 2014). To date, more than 200 SOD1 mutations have been documented, with the majority being missense mutations. Other variants are seen in those with varying rates of disease progression (Elmansy et al., 2023), which is one of the reasons for the high heterogeneity of ALS. The mechanisms by which mutant SOD1 leads to cell death are not clear; however, some experts have demonstrated that it results in a toxic gain of function (GOF). Mutant SOD1 may induce motor neuron death through reactive oxygen species (ROS), excitotoxicity, mitochondrial dysfunction, axonal transport disruption, and the autonomous non-cell toxicity of neuroglia (Hayashi et al., 2016). Thus, inhibiting the production of mutant SOD1 or promoting its degradation is a potential therapeutic approach for ALS (Bunton-Stasyshyn et al., 2015). Numerous studies using gene silencing mediated by antisense nucleotides, RNA interference, microRNA (miRNA), small-interfering RNA (siRNA), short-hairpin RNA, and CRISPR/Cas9 gene editing have been conducted (Reddy and Miller, 2015; Yun and Ha, 2020; Amado and Davidson, 2021; Van Daele et al., 2024). Such techniques have demonstrated certain advantages, and these studies offer references for the continuous improvement of therapies that target SOD1 in the future (Abati et al., 2020).

In addition to GOF, there have been reports of loss of function (LOF) mutants of SOD1 in ALS. For example, the symptoms observed in patients with a homozygosity of truncated SOD1 proteins are significantly more severe than heterozygous carriers who retain some SOD activity. This indicates that the incomplete knockdown of SOD1 and use of nanoengineered delivery tools may be reasonable and safe options for ALS treatment, offering new ideas for further clinical trials (Meijboom and Brown, 2022).

#### Chromosome 9 open reading frame 72 gene

The *C9orf72* mutation is characterized by the presence of a hexanucleotide (GGGGCC) repeat expansion (HRE) in *C9orf72*, and it has been associated with ALS. Currently, C9orf72 is the most prevalent genetic mutation in ALS patients of Caucasian origin, accounting for 40% of fALS cases and 7% of sALS cases (Elmansy et al., 2023). Healthy individuals typically have fewer than 30 repeats, whereas those with C9orf72-ALS may exhibit hundreds to thousands of abnormal repeats (Rohrer et al., 2015). The HRE results in a toxic GOF effect, leading to the impaired transcription and production of toxic-aggregation-prone dipeptides. Moreover, the HRE promotes the formation of RNA foci, which when combined with RNA-binding proteins, exerts adverse effects on RNA expression and splicing (Kim et al., 2020). These mechanisms offer feasible targets for drug development, and the further development of highly sensitive approaches for the precise quantification of RNA repeats and toxic dipeptide repeats could be warranted. Alternatively, HRE in *C9orf72* can also cause LOF, as a recent clinical study (van den Berg et al., 2024) showed that an antisense oligonucleotide drug BIIB078, which binds to a site in intron 1 of *C9orf72*, resulted in the degradation of sense RNA containing hexanucleotide repeats transcribed from mutant *C9orf72* via RNase H and a reduction in toxic dipeptides. However, the final study results were not sufficient to achieve the inhibition of amplification toxicity and thus not properly solve the problem mediated by downstream dysfunction.

Among the 28 trialed drugs, LAM-002A/Apilimod dimesylate (NCT05163886) (Babu et al., 2024) and TPN-101 (NCT04993755) were designed for patients with the *C9orf72* mutation (**Table 1**). Fortunately, the former, a phase 2a clinical trial, showed promising results suggesting Apilimod dimesylate might have an impact on all forms of ALS, not merely C9orf72-ALS, and might reverse the prevalent TDP-43-associated pathology. Although these are the only two trials to deal with a defined population of targeted mutations, the outcomes of LAM-002A also elucidated the scientific utility of targeting C9orf72 as the most common mutation and the generalizable effects of potential accompanying mechanisms.

**Table 1:** DMT drugs in ALS

Name	Target class	Specific target	Mechanism	Phase	Period	IDs	Route	Country
SLS-005τ	Anti-aggregation	activation of TFEB	As an mTOR-independent inducer of autophagy, it can facilitate TDP-43 clearance by activating the autophagic degradation pathway.	2/3	02.2022–08.2023	NCT05136885	Infusion	USA
LAM-002Aτ@	Anti-aggregation	PIKfyve kinase inhibitor 1,2	As the inhibitor of the lipid kinase PIKfyve, lead to activation of the transcription factor TFEB which drives the increased clearance of toxic protein aggregates via the autophagy lysosomal pathway.	2	12.2021–01.2023	NCT05163886	Oral	USA
ABBV-CLS-7262τ	Anti-aggregation	UPR PERK	Restore protein production, clear TDP-43 aggregates, and improve the survival of nerve cells by activating EIF2B.	1	09.2021–02.2023	NCT04948645	Oral	USA
Bosutinib	Anti-aggregation	Scr-c/Abl inhibitor	As a Scr-c/Abl inhibitor, it improves autophagy to decrease misfolded proteins accumulation.	1/2	03.2019–03.2024	NCT04744532	Oral	Japan
AMX0035	Anti-apoptotic	Mitigate endoplasmic reticulum stress and mitochondrial dysfunction	Simultaneously, mitigate endoplasmic reticulum (ER) stress and mitochondrial dysfunction.	3	10.2021–11.2023	NCT05021536	Oral	USA
TUDCA (Tauroursodeoxycholic Acid)	Anti-apoptotic	Mitigate endoplasmic reticulum stress and mitochondrial dysfunction	Exact mechanism is unknown; believed to decrease neuronal cell death by decreasing ER stress and mitochondrial dysfunction	3	02.2019–12.2023	NCT03800524	Oral	Belgium, France
Enoxacin	Anti-apoptotic	Enhance DICER activity as a small-molecule enhancer of microRNA (SMER) maturation	Mitigate stress enhancing DICER activity by a small molecule	1/2	03.2021–05.2023	NCT04840823	Oral	Canada
PrimeC	Anti-neuroinflammation	COX2 inhibitor	Potentially interfer with glutamate-induced excitotoxicity, inflammation, and oxidative stress (OS)-related toxicity	2	05.2022–11.2023	NCT05357950	Oral	Canada, Israel, Italy
MediCabilis CBD Oil	Anti-neuroinflammation	CB1, CB2 inhibitor	Strong antioxidative and neuroprotective effects, which may prolong neuronal cell survival.	3	01.2019–12.2023	NCT03690791	Oral	Australia
TPN-101@	Anti-neuroinflammation	Long interspersed nuclear element 1 (LINE1) reverse transcriptase	By inhibiting LINE1 reverse transcriptase, TPN-101 is expected to prevent this immune response and protect cells.	2	10.2021–09.2023	NCT04993755	Oral	USA
ANX005	Anti-neuroinflammation	C1q inhibitor	A humanized immunoglobulin G4 recombinant antibody against C1q that inhibits its function as the initiating molecule of the classical complement cascade for neuroprotection.	2	01.2021–10.2023	NCT04569435	Injection	USA
MN-166 (Ibudilast)τ	Anti-neuroinflammation	Macrophage migration inhibitory factor (MIF) and phosphodiesterases 3,4,10 and 11	Inhibition of proinflammatory cytokines and macrophage migration inhibitory factor. Increasing autophagy against pathogenic TDP-43-induced cytotoxicity by inhibiting the mTORC1 activity.	2/3	05.2020–12.2023	NCT04057898	Oral	USA
Masitinib	Anti-neuroinflammation	A tyrosine kinase inhibitor	Modulate the neuroinflammatory activity of ALS by targeting macrophages, mast cells, and microglia cells.	3	02.2021–12.2023	NCT03127267	Administered subcutaneously	USA
Fasudil (WP-0512)	Anti-neuroinflammation	Rho kinase (ROCK) inhibitor	Improve the regenerative response in the injured central nervous system and neuronal survival, including the promotion of neuromuscular junction maturation.	2	12.2021–07.2023	NCT05218668	Oral	USA
FC-12738	Anti-neuroinflammation	Mimic thymopentin 5	FC-12738 activates toll-like receptors 2, regulating cytokines and inflammatory mediators to mitigate neuroinflammation.	1	03.2024–06.2024	NCT05978908	Subcutaneous injection	USA
Deferiprone	Anti-oxidative stress	Iron in the brain	Scavenge iron from labile iron complexes in the brain and transferred (conservatively) either to higher affinity acceptors in cells or extracellular transferrin, reducing focal iron accumulation associated with brain iron.	2/3	02.2019–11.2023	NCT03293069	Oral	France
Antioxidants#	Anti-oxidative stress	/	/	2	04.2022–12.2023	NCT04244630	Oral	USA
MT-1186 (Edaravone)*τ	Anti-oxidative stress	Scavenge reactive oxygen species (ROS)	As a free radical scavenger: eliminate lipid peroxides and hydroxyl radicals to mitigate oxidative injury in neurons, preventing TDP-43 misfolding and enhancing clearance of pathological TDP-43 by reducing apoptosis and ER stress	3	10.2022–09.2023	NCT05568615	Oral	Japan
MT-1186*τ	/	/	/	3	11.2020–09.2023	NCT04569084	Oral	USA
MT-1186*τ	/	/	/	3	10.2020–09.2023	NCT04577404	Oral	USA
FAB122	Anti-oxidative stress	Scavenge reactive oxygen species (ROS)	As a free radical scavenger: eliminate lipid peroxides and hydroxyl radicals to mitigate oxidative injury in neurons.	3	10.2021–06.2024	NCT05178810	Oral	France
Trimetazidine Dihydrochloride	Metabolism	A metabolic modulator	Enhance mitochondrial metabolism and promote nerve regeneration, exerting an anti-inflammatory and antioxidant effect.	2	06.2021–05.2023	NCT04788745	Oral	Australia, Netherlands, UK
CNMAu8	Metabolism	Catalyze for energy metabolism	An aqueous suspension of clean-surfaced, faceted gold nanocrystals are shown to be an efficient catalyst for energy metabolism.	2	11.2021–11.2023	NCT05299658	Oral	Australia
Ambroxol	Metabolism	Inhibit GBA2	Neuroprotective effects by modulating glucocerebrosidase activity. Stimulate axonal plasticity and motor recovery.	2	06.2023–06.2024	NCT05959850	Oral	Australia

# Including vitamin E, NAc cysteine, L-cystine, nicotinamide and taurursodiol; * the same drug in different trials; τ drugs related to TDP-43-reduced therapy; @ drugs aimed at the C9orf72 mutant population; the remainder are for general ALS. ALS: Amyotrophic lateral sclerosis; CB2: cannabinoids 2; DMT: disease-modifying therapy.

#### TAR DNA binding protein 43

TARDBP, which encodes TDP-43, was first identified as an ALS-related gene in 2006. Mutant TDP-43 accounts for in 4% of fALS and 1% of sALS cases, while pathological TDP-43 can be detected in the brain or spinal cord in over 95% of ALS cases and over 40% of frontotemporal dementia cases (Prasad et al., 2019). TARDBP variants lead to the cytoplasmic aggregation and mis-localization of TDP-43, resulting in both GOF and LOF effects, such as RNA metabolism disorders, splicing defects, impaired mitochondrial function and axon transport, protein balance defects, stress granule formation, and amyloid aggregation (Meijboom and Brown, 2022; Akçimen et al., 2023). Aberrant TDP-43 is regarded as a significant marker of ALS. To prevent disease progression, many therapeutics aim to eliminate harmful microenvironmental elements, and therapies aimed at TDP-43 inclusion removal and prevention and the restoration of TDP-43 regulation can improve cellular function, slowing or halting the disease. Thus, multimodal treatments should be evaluated for TDP-43 proteinopathy (Ho et al., 2024).

Among the drugs investigated, SLS-005 (NCT05136885), LAM-002A (NCT05163886), ABBV-CLS-7262 (NCT04948645), MN-166/Ibudilast (NCT04057898), and MT-1186/Edaravone (NCT05568615; NCT04569084; NCT04577404), were found to directly target TDP-43 clearance (**Table 1**), accounting for approximately 20% of all the therapeutics. This underlines the potential of targeting TDP-43 as a prominent research and development orientation. Although other drugs, such as masitinib, TPN-101, and enoxacin, also induce alterations in TDP-43 levels, their specific mechanisms remain ambiguous, and they do not directly target TDP-43 clearance. Furthermore, CNS10-NPC-GDNF and lenzumestrocel are stem cell therapies that can lower TDP-43 levels; however, they do not belong to the category of targeted therapy drugs. While these treatments might reduce the levels of TDP-43 through indirect mechanisms and enhance the neuron count and lifespan, they were excluded from the statistical analysis. Each drug encompassed by this analysis functions through disparate mechanisms but ultimately intends to diminish or eliminate the production and/or accumulation of TDP-43 (Wang et al., 2018; Chen et al., 2020; Hung et al., 2023).

Since 2018, SLS-005 (also known as trehalose) has been demonstrated to independently induce autophagy and facilitate the clearance of TDP-43 through the activation of the autophagic degradation pathway and transcription factor EB (TFEB)-mediated transcriptional activity (Wang et al., 2018). The clinical trial challenged the conventional use of an oral administration mode for trehalose, as it showed its safety and efficacy when administered in a modified dosage form via intravenous injection, presenting a repurposing strategy for trehalose. LAM-002A is a potent and highly selective inhibitor of the lipid kinase PIKfyve, which also induces the activation of the transcription factor TFEB and therefore accelerates the clearance rate of toxic protein aggregates through the autophagy-lysosomal pathway (Hung et al., 2023). By inhibiting PIKfyve kinase activity, LAM-002A promotes lysosome biogenesis, resulting in the efficient elimination of existing toxic protein aggregates and preventing their further accumulation. MN-166 also enhances the clearance of TDP-43 protein aggregates by augmenting TFEB-dependent lysosomal activity and abundance (Chen et al., 2020). ABBV-CLS-7262, an orally administered compound, operates by activating the eIF2B protein complex, which is indispensable for efficient protein synthesis. This complex is suppressed during stress responses, as it is a component of the integrated stress response, which restores the cellular and proteostasis equilibrium to prevent cell death (Szewczyk et al., 2023). By activating eIF2B, it is anticipated that ABBV-CLS-7262 can restore protein synthesis, eliminate TDP-43 aggregates, and enhance the viability of neuronal cells. Indeed, this treatment has demonstrated an ability to restore protein production and disintegrate preformed stress granules containing TDP-43 (Bugallo et al., 2020). MT-1186 is an orally administered formulation of edaravone, which was previously approved only for intravenous administration. The statistical analysis encompassed three studies that investigated the safety and efficacy of the oral edaravone suspension. The results indicated that edaravone elicits significant modifications in gene expression within the unfolded protein response and autophagy pathways in TDP-43-expressing cells. These findings suggest that the neuroprotective effects of edaravone encompass its inhibition of TDP-43 misfolding and its enhancement of pathological TDP-43 clearance in TDP-43 proteinopathies, evidencing the broad applicability of edaravone (Soejima-Kusunoki et al., 2022).

#### Fused in sarcoma

In 2009, a newly discovered pathological mutation in the *FUS* gene was identified in ALS. *FUS* variants, occurring in 5% of fALS and 1% of sALS cases, are typically found in patients with early-onset and juvenile ALS (Elmansy et al., 2023). Similar to TDP43, FUS regulates gene expression by controlling transcription, translation, and RNA transport (Vance et al., 2009). In addition, FUS is involved in DNA repair through homologous recombination and non-homologous end joining (Wang et al., 2013). Missense mutations of FUS can lead to a toxic GOF or LOF effect, the mechanism of which is still under debate and requires further research. Several results suggest that FUS LOF alone does not cause FUS-ALS but might lead to other deleterious phenotypes (Kim et al., 2020). Analogous to the pathogenesis of TDP-43, pathological FUS forms cytoplasmic aggregates and mis-localizations composed of RNA and various proteins that impair cellular functions; therefore, therapeutic methods targeting FUS are a promising direction.

### Proposed amyotrophic lateral sclerosis pathways

**Figure 2:**
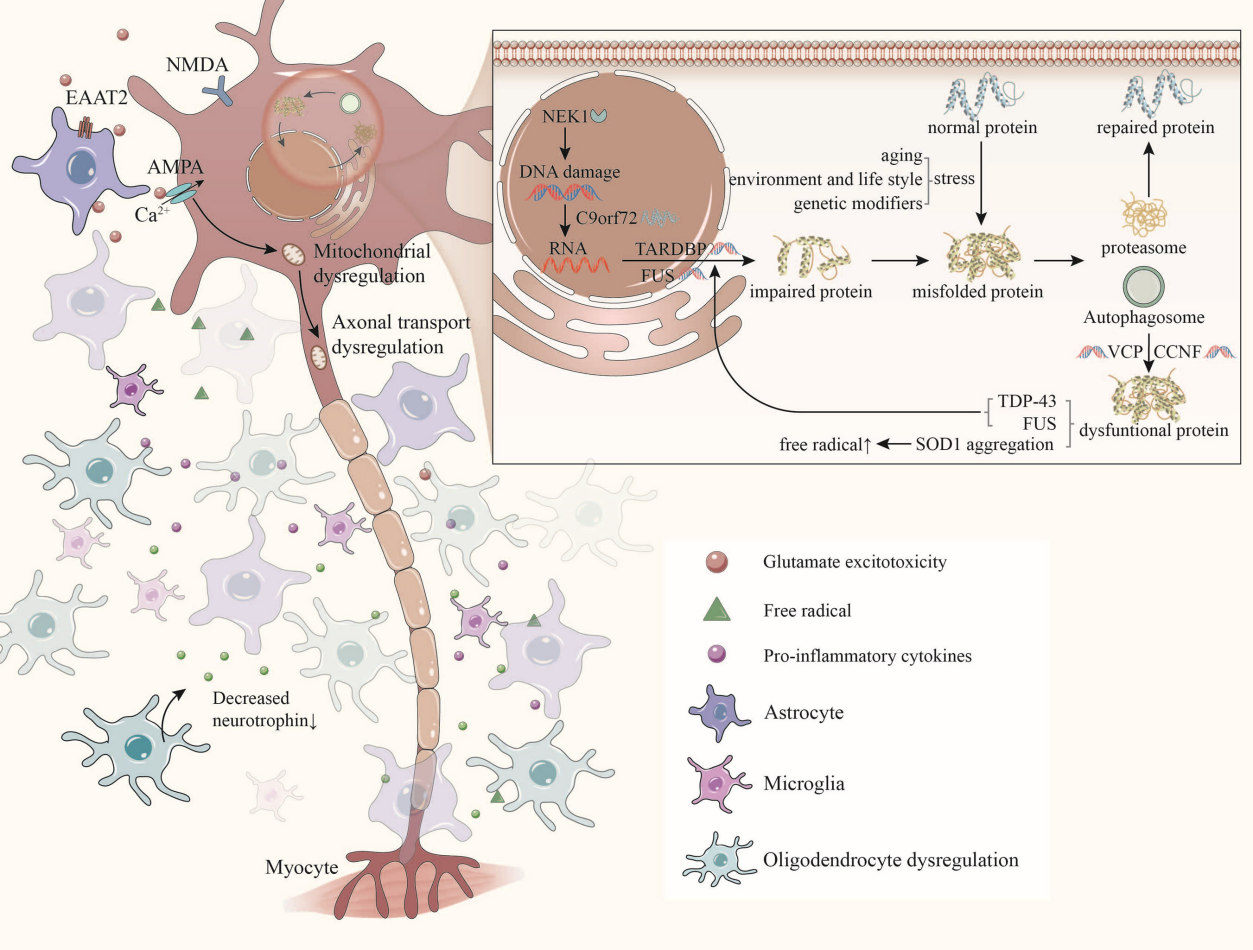
Major pathogenesis of ALS. The pathological changes in motor neurons in ALS patients are partly regulated by mutant genes, and several common pathological mechanisms have also been reported. Among them, C9orf72 repeats may cause a toxic gain of function, thereby affecting transcription and leading to the aggregation of toxic proteins. NEK1 loss-of-function (LOF) mutations affect the DNA damage response, leading to disturbances at the DNA level. TARDBP and FUS mutations affect mainly the transcription process of RNA. In addition to primary gene mutations, there are multiple combined factors involved in the pathogenesis of ALS, including age, living habits and genetic predispositions to some related phenotypes. Stress can cause protein levels to be disordered, and healthy people can be cleared and corrected by the proteasome and autophagosomes; however, in some patients with VCP and CCNF gene mutations, this link function is impaired. This results in the production and aggregation of aberrant functional proteins. In addition, glial cells are involved in pathological mechanisms. The impaired function of EAAT2 receptors on astrocytes leads to glutamate reuptake disorders, which continuously act on AMPA and NMDA receptors on motor neurons to produce excitotoxic effects. This leads to a large amount of calcium ion influx, resulting in mitochondrial calcium overload energy, thus affecting axonal transport. SOD1 gene mutation leads to decreased antioxidative stress function, damaging nerve cells via free radicals produced by microglia. The production of proinflammatory factors after activation by inflammasomes can also perturb the function of nerve cells. Oligodendrocytes are also dysfunctional and thus produce fewer neurotrophins. ALS: Amyotrophic lateral sclerosis; AMPA: α-amino-3-hydroxy-5-methyl-4-isoxazolepropionic acid; CCNF: cyclin F; EAAT2: excitatory amino acid transporter 2; FUS: fused in sarcoma; NMDA: N-methyl-D-aspartate; SOD1: superoxide dismutase 1; TARDBP: TAR DNA-binding protein; TDP-43: transactive response DNA-binding protein 43; VCP: valosin-containing protein.

The pathogenesis of ALS is not attributable to a single pathway, but rather it results from the interplay of multiple pathways, leading to progressive cellular damage and eventual necrosis or apoptosis (**Figure 2**). Oxidative stress arises from the excessive accumulation of ROS and reactive nitrogen species, as well as impairments to the body’s antioxidant system. Apart from directly damaging the cellular structure, oxidative stress can also expedite the deterioration of other pathological pathways, such as facilitating the aggregation of TDP-43 and release of the excitatory neurotransmitter glutamate (Cohen et al., 2015; Kazama et al., 2020). Excitotoxicity arises from an elevation in glutamate concentration, leading to the excessive activation of postsynaptic glutamate receptors and thus heightened neuronal discharge and elevated intracellular calcium ions, ultimately triggering endoplasmic reticulum stress and mitochondrial calcium overload (Mead et al., 2023). Furthermore, elevated levels of glutamic acid can diminish the concentration of the antioxidant cysteine and worsen cell damage by inhibiting its reverse transport (Lewerenz et al., 2013). Excitotoxicity results in mitochondrial calcium overload, which promotes mitochondrial dysfunction. The disruption of mitochondrial function can impact energy production, lead to the excessive generation of ROS, disrupt mitochondrial axon transport, and even induce cell apoptosis (Debska-Vielhaber et al., 2021). Mitochondria, as cellular energy supply stations, also play a role in metabolism when their function is impaired. More significantly, when the energy supply is terminated or impaired, protein synthesis in the physiological state is disturbed, leading to an imbalance in protein homeostasis. The maintenance of intracellular protein homeostasis relies on cell synthesis, transport, proteases, autophagy systems, and other factors. Any disruptions in these pathways that result in a protein imbalance, such as the accumulation of misfolded proteins, may trigger cellular aging and contribute to age-related neurodegenerative diseases (Di Domenico and Lanzillotta, 2022). In addition to these mechanisms, neuroinflammation also plays a crucial role in ALS. For instance, the activation of astrocytes leads to the release of inflammatory mediators such as prostaglandins and leukotriene (Hensley et al., 2006). Furthermore, the complement system, with an increased level of C1q or C5, contributes to motor neuron death by forming the membrane attack complex and recruiting monocytes. Additionally, the infiltration of the spinal cord by lymphocytes is implicated in motor neuron degeneration (Liu and Wang, 2017). It is a commonly held belief that the exposure of genetically susceptible individuals to adverse environments, such as pesticides and heavy metals, ultimately gives rise to the occurrence of degenerative MNDs. That is, the combination of genetic and environmental factors contributes to their emergence. It has been proposed, in the gene-time-environment hypothesis of ALS, that even carriers of ALS mutations require several stimuli, possibly from environmental exposures, to trigger the disease (Goutman et al., 2023). There is no denying that the discovery of the pathophysiological mechanisms of the disease helps us to develop targeted drugs, and thereby improve drug efficacy.

## Drugs

### Approved drugs

**Figure 3:**
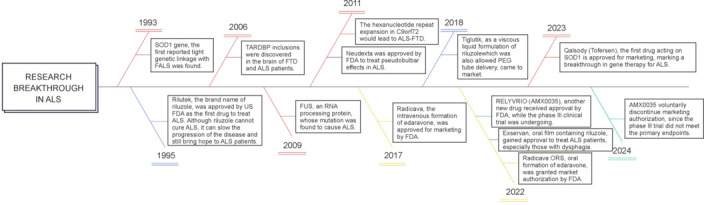
Research breakthroughs in ALS. This figure illustrates the chronological sequence of the causative genes investigated in major ALS studies, along with the current status of the approved drugs. Created with X-mind. ALS: Amyotrophic lateral sclerosis; FALS: family amyotrophic lateral sclerosis; FTD: frontotemporal dementia; ORS: oral suspension; PEG: percutaneous endoscopic gastrostomy; SOD1: superoxide dismutase 1; TARDBP: TAR DNA-binding protein.

Five drugs have received approval from the U.S. FDA for delaying the progression of ALS: Qalsody, riluzole, edaravone, Relyvrio, and Nuedexta (**Table 2**). Three formulations of riluzole and two formulations of edaravone were derived to resolve difficulties encountered in the application of the drugs. The fishbone diagram provided in **Figure 3** of the major researched genes and approved drugs for ALS illustrates the challenges and complexities encountered in the development of treatments for ALS.

**Table 2:** FDA-approved drugs for ALS

Drug	DMT									Non-DMT
		
	Riluzole				Edaravone		Relyvrio	Qalsody		Nuedexta
		
	Rilutek	Tiglutik	Exservan		Radicava	Radicava ORS				
Target Class	Anti-excitotoxicity				Anti-oxidative stress		Anti-apoptotic	Anti-aggregation		\
Mechanism	Glutamatergic neurotransmission; stimulate BDNF and modulate sodium channels.				Eliminate lipid peroxides and hydroxyl radicals to mitigate oxidative injury in neurons.		Simultaneously, mitigate endoplasmic reticulum (ER) stress and mitochondrial dysfunction.	An antisense oligonucleotide designed to reduce the synthesis of SOD1 protein by inducing RNase H–mediated degradation of SOD1 messenger RNA.		Activate sigma-1 receptor (S1R) to regulate signal molecular and ion channels.
Target population	General				General		General	SOD1 Mutants		General
Route	Oral				Injection	Oral	Oral	Injection		Oral
First approved year	1995	2018	2022		2017	2022	2022	2023		2011
Country	USA				USA		USA	USA		USA

ALS: Amyotrophic lateral sclerosis; DMT: disease-modifying therapy; FAD: the US Food and Drug Administration; ORS: oral suspension.

#### Qalsody

Qalsody, also known as tofersen or BIIB067, is an antisense oligonucleotide (ASO) that can be administered via lumbar puncture to overcome the challenges posed by the blood–brain barrier and effectively deliver treatment. It is a small strand of DNA designed to induce the RNase H-mediated degradation of *SOD1* mRNA and thereby reduce SOD1 protein synthesis (Miller et al., 2022). Studies have shown that ASO can also decrease neurofilament light chain (Nfl) concentrations in cerebrospinal fluid and serum, leading to improved ALS progression and survival (Meyer et al., 2023). On April 25, 2023, Qalsody received FDA approved for treating SOD1-ALS patients, making it the latest drug authorized for ALS therapy (Blair, 2023).

*SOD1* mutations are the second most prevalent cause of fALS, accounting for approximately 20% of fALS cases and 1%–2% of sALS cases (Abati et al., 2020; Brown et al., 2021). SOD1 plays a crucial role in breaking down the toxic byproducts generated during normal cellular processes. Mutant SOD1 undergoes misfolding and aggregation within motor neurons and astrocytes (Peggion et al., 2022), potentially disrupting nerve cell function or inducing the misfolding and LOF of other essential proteins, ultimately leading to neuronal degeneration and promoting ALS development (Abati et al., 2020). Initially, ASOs were reported to modulate RNA processing and protein expression. Therefore, with recent breakthroughs in chemical modifications, the inherent limitations of ASOs, including their suboptimal bioactivity, insufficient target engagement, and off-target toxicity, have been successfully mitigated. These advancements have subsequently facilitated the transition of this class of therapeutic agents from the research realm into clinical application (Rinaldi and Wood, 2018). Over the past few years, four antisense drugs, eteplirsen, golodirsen, viltolarsen, and casimersen, have respectively received FDA approval as treatments for Duchenne muscular dystrophy, and nusinersen has been approved for spinal muscular atrophy (SMA), bolstering our confidence in the utility of antisense drugs in ALS treatment (Brunet de Courssou et al., 2021). The research into these drugs also provides insights into how to treat patients with other mutant genes, such as *C9orf72*, with antisense nucleotides. However, commonly reported adverse reactions of tofersen treatment include back pain, headache, neck pain, extremity pain, and cerebrospinal fluid pleocytosis, primarily associated with lumbar puncture (Miller et al., 2020). Therefore, the inconvenience posed by this invasive delivery method remains a significant obstacle hindering tofersen’s clinical application, which necessitates further improvements (Everett and Bucelli, 2024).

#### Riluzole

Rilutek, Tiglutik, and Exservan are all brands containing riluzole, which exerts inhibitory effects on glutamate release and voltage-dependent calcium channels to counteract the intercellular damage caused by the excitatory neurotransmitter glutamate (Cheah et al., 2010). Rilutek was the first drug to receive FDA approval for ALS treatment, which it received in 1995. It is a white film-coated tablet that is administered orally. However, in practical applications, many advanced ALS patients experience dysphagia due to facial and throat muscle weakness, which is often accompanied by difficulties with chewing, salivation, speaking, and drinking. To address the dysphagia challenges, numerous ALS patients resort to grinding their pills; however, this practice may result in medication loss or a reduction in efficacy while also posing risks of choking and aspiration (Borasio, 2002). Therefore, Tiglutik was formulated as a viscous liquid riluzole, enabling its oral administration via syringe and mitigating the risks and challenges associated with the oral tablets mentioned above. It is also the sole approved riluzole formulation for PEG tube delivery, helping to reduce treatment interruptions caused by difficulties with swallowing tablets or the use of feeding tubes. Its slightly thickened consistency facilitates a slower drug flow and increases drug utilization (Romero-Gangonells et al., 2023). Similarly, Exservan was developed specifically for individuals with severe dysphagia to address the medication challenges arising from patients’ swallowing problems. This innovative formulation incorporates oral film technology, allowing the drug to be placed on the patient’s tongue for dissolution. Absorption occurs through dense capillaries beneath the sublingual or buccal mucosa. A rapid onset of action is ensured due to the drug’s ease in penetrating these tissues without causing harm to normal tissue, while its degradation in the acidic and enzymatic environment of the gastrointestinal tract is avoided (Qureshi et al., 2020). The updated iterations of various dosage forms of riluzole have also prompted consideration regarding the development of drugs for rare diseases. Given that certain drugs can potentially delay disease progression, it becomes imperative to address the inherent physical limitations reasonably and promptly in order to enhance drug utilization efficiency (Ludolph et al., 2023). Naturally, certain unpredictable drug defects necessitate continuous explorations through experiments, but it is a race against time to help patients.

#### Relyvrio

Relyvrio (AMX0035) is a combination of sodium benzoate and taurine diol. Several studies have demonstrated the diverse biological effects of sodium benzoate, with evidence suggesting that it can modulate ALS progression in cell models by altering transcription, reducing neuroinflammation, and improving cellular energy metabolism (Sun et al., 2022). Additionally, as a histone deacetylase inhibitor, sodium benzoate can attenuate the endoplasmic reticulum stress response (Zhang et al., 2017). Taurine diol exhibits anti-apoptotic and anti-inflammatory properties through its inhibition of mitochondrial membrane permeability (Rodrigues et al., 2003), which represents a potential mechanism for slowing down ALS progression. In a phase 2 trial (Sun et al., 2023), relyvrio reduced the levels of markers, such as Tau and YKL-40, associated with neurodegenerative diseases, while achieving its primary clinical endpoint (Paganoni et al., 2022). Given the urgent need for therapeutic interventions in ALS disease, AMX0035 was expected to receive marketing approval in 2022. However, based on the results of the phase 3 PHOENIX trial, the efficacy of this drug remains uncertain, leading to its voluntary withdrawal from both US and Canadian markets. Although the limited availability of short-term survival has posed challenges during clinical trials, it cannot be denied that positive outcomes from the phase 2 trial played a significant role in obtaining approval. This withdrawal also reflects the difficulty and urgency of drug development for rare diseases. Each drug approval is not only good news for patients but also an opportunity to promote the continuous improvement of traditional drugs and the innovation of new ones to attain greater universal benefits.

#### Edaravone

As a potent scavenger of free radicals, edaravone exhibits the ability to mitigate oxidative stress and impede disease progression while conferring a cytoprotective effect on nerve cells to safeguard against neuronal degeneration (Cho and Shukla, 2020). Radicava, an intravenous formulation of edaravone that gained approved in 2017, has been demonstrated by multiple studies to exhibit selective efficacy for certain patients with highly heterogeneous ALS (Shefner et al., 2020; Witzel et al., 2022). To overcome the limitations associated with its intravenous administration, Radicava ORS (edaravone) presents an oral alternative that enables convenient self-administration at home or via tube feeding.

A previous study (Jaiswal, 2019) demonstrated that there exist remarkable differences and scant similarities among the mechanisms of action of riluzole and edaravone. Riluzole mainly interferes with the excitotoxic pathway regulated by glutamine, whereas edaravone benefits patients by scavenging free radicals; yet both are neuroprotective. The substantial dissimilarity between these two approved drugs not only reflects the complexity of ALS but also alerts us of the varying genotypic characteristics of ALS subtypes, as the same ALS mutation may present as different phenotypes. The same drug is not necessarily applicable to everyone, and personalized treatment constitutes one of the goals of drug development.

#### Nuedexta

The FDA approved Nuedexta in 2011 as a therapeutic option for ALS patients suffering from the pseudobulbar affect, a common symptom characterized by frequent, involuntary, and often sudden episodes of exaggerated crying and/or laughing that are inconsistent with the individual’s true emotions (Sun et al., 2024). However, it should be noted that Nuedexta does not have any impact on disease progression.

### Clinical drugs

**Figure 4:**
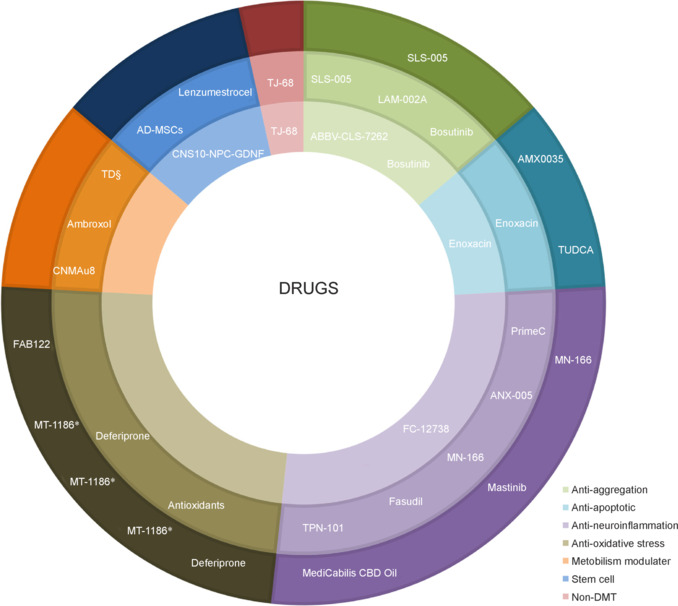
Classification of drug stages and mechanisms. The pie chart is divided into three layers: the inner layer represents phase 1, the outer layer represents phase 3, and the middle layer represents phase 2. Owing to certain drugs being marked in multiple phases, it is possible for a single drug to appear in different layers. Additionally, according to their mechanisms of action, all the drugs were classified into seven categories: anti-aggregation, anti-apoptotic, anti-neuroinflammatory, anti-oxidative stress, metabolism modulator, stem cell, and non-DMT. * the same drug in different trials; § short for Trimetazidine Dihydrochloride. AD-MSCS: adipose-derived mesenchymal stem/stromal cells; AMX0035: sodium phenylbutyrate-taurursodiol combination; ANX-005: anti-human complement C1q antibody; CNS10-NPC-GDNF: CNS10 neural progenitor cells engineered to secrete glial cell line-derived neurotrophic factor; FAB122: oral edaravone; LAM-002A: liposomal annonaceous acetogenin-002A; MN-166: ibudilast; MT-1186: edaravone oral suspension; non-DMT: non-disease-modifying therapy. Prime C: combination of ceftriaxone and ciprofloxacin with additional neuroprotective agents; SLS- 005: sodium phenylbutyrate-taurursodiol; TJ-68: shakuyakukanzoto (a kind of Kampo medicine); TPN-101: prasinezumab; TUDCA: tauroursodeoxycholic acid.

As detailed in the search strategy section, 28 drugs were included in the analysis: 24 were DMT, 3 were stem-cell agents, and 1 was non-disease-modifying therapy (nDMT). It should be mentioned that two additional drugs were excluded from the analysis, namely Pegcetacoplan/APL-2 (NCT04579666) and WVE-004 (NCT04931862), as they had been terminated for various reasons, which we determined through searching other databases. The former was approved by the FDA in 2021 for the treatment of paroxysmal nocturnal hemoglobinuria and received approval in 2023 for the treatment of geographical atrophy secondary to age-related macular degeneration. In a phase 2 re-exploration trial, it indicated its ability to serve as a complement C3 inhibitor for ALS treatment, but owing to a combination of funding issues and unsatisfactory outcomes, the trial was automatically abandoned. However, the website did not provide updates on the status of the trial, not only augmenting the difficulty of obtaining data for statistics but also revealing the austere situation of drug research and development. The latter, WVE-004, a stereopure oligonucleotide for the treatment of C9ORF72-related ALS, showed promising activity for reducing toxic protein levels in preclinical and phase 1/2 studies. Nevertheless, no improvements in functional status were observed in patients in the FOCUS-C9 trial (Liu et al., 2022). Consequently, it was unfortunately discontinued due to the insignificant clinical benefits. All included drugs were preliminarily classified into three categories according to their stage of clinical trial: phases 1, 2, and 3. Then, based on their mechanisms of action, they were further categorized into seven groups, as depicted in **Figure 4** (Jiang et al., 2022; Johnson et al., 2022).

#### Disease-modifying therapy drugs

We compiled a collection of 24 DMTs that have demonstrated potential in decelerating the progression of the disease. **Table 1** presents a comprehensive overview of information pertaining to all clinical DMT drugs. On inspection of **Table 1** in conjunction with **Figure 4**, it is not arduous to observe that those targeting anti-inflammatory and antioxidant mechanisms constitute the largest proportion of drugs, which is confluent with the significant roles of these two mechanisms in the pathogenesis of ALS. Additionally, most of the drugs are in phases 2 and 3, and thus have demonstrated a certain amount of potential. MT-1186 is particularly promising and is undergoing three simultaneous phase 3 trials. MT-1186, which is an oral version of edaravone, was granted market approval in 2022 on account of its notable clinical benefit. Nevertheless, since its approval, studies assessing its efficacy in comparison with intravenous formulations have persisted. Moreover, it offers a clinically superior alternative for patients due to its oral suspension mode of administration, which provides a less cumbersome option compared to the intravenous administration of the previously approved Radicava, and it features a shorter administration duration. It can be taken orally or through a feeding tube without dose adjustment and can be stored at room temperature, enhancing its portability. Furthermore, its safety and tolerability still require prolonged testing, thus more clinical trials will be indispensable following its approval.

*Anti-aggregation*

In the anti-aggregation category, we collected data on four drugs: ABBV-CLS-7262 is currently in phase 1 clinical trials, bosutinib is in phase 1/2, LAM-002 is in a phase 2 clinical trial, and SLS-005 is undergoing a combined phase 2/3 clinical trial. By inducing the translocation of TFEB (Rusmini et al., 2019), SLS-005 facilitates the activation of a specific sequence (E-box) on the promoter of an autophagy gene (Napolitano and Ballabio, 2016), thereby regulating the lysosomal and autophagy pathways (Franco-Juárez et al., 2022). In the latest clinical trials, the Src/c-Abl inhibitor bosutinib was shown to decrease pathological protein accumulation by enhancing autophagy (Imamura et al., 2017), and a previous phase 1 trial confirmed its safety and efficacy (Imamura et al., 2022). The remaining two drugs target alternative mechanisms, such as cell transmembrane transport. For instance, LAM-002 functions as a PIKfyve inhibitor in C9orf72-ALS pluripotent stem-cell-induced motor neurons to enhance the exocytosis of pathological and neurotoxic proteins (Crunkhorn, 2023; Hung et al., 2023). Whereas ABBV-CLS-7262 is a novel compound designed to degrade stress response granules containing TDP-43 and thus inhibit the integrated stress response in neurons (Luan et al., 2023).

*Anti-apoptotic compounds*

There are three ongoing clinical trials investigating anti-apoptotic agents: one drug (enoxacin) is in a phase 1/2 trial and two (AMX0035, TUDCA) are in phase 3 trials. AMX0035 has been demonstrated to modulate mitochondrial membrane perturbation and alleviate ER stress, thereby safeguarding cells against apoptosis (Rodrigues et al., 2003; Lynch, 2023). TUDCA, similar to AMX0035, was demonstrated in a previous phase 2 clinical trial to slow disease progression (Elia et al., 2016). Double-stranded RNA-specific endoribonuclease (DICER) is a conservative protein involved in various processes, including apoptosis, transcription, DNA repair, and autophagy (Vergani-Junior et al., 2021). The stress response negatively impacts DICER function. In ALS, the combination of stress response granules and DICER leads to the dysfunction of DICER itself. Enoxacin can enhance DICER activity and maintain its intracellular functions (Emde et al., 2015).

*Anti-neuroinflammation compounds*

Neuroinflammation is the primary target of many new drugs developed for ALS, accounting for the largest proportion of ALS drugs being investigated (33%). Of these new drugs, one is in a phase 1 clinical trial (FC-12738), four are in phase 2 clinical trials (PrimeC, TPN-101, ANX005, and Fasudil), two are in phase 3 clinical trials (MediCabilis CBD Oil, Masitinib), and one is in a combined phase 2/3 clinical trial (MN-166). Masitinib inhibits tyrosine kinase to reduce immune cell production and activation as a strategy to mitigate inflammation. Additionally, it specifically targets macrophages, mast cells, and microglia to alleviate neuroinflammation within the nervous system (Trias et al., 2016; Mora et al., 2020). ANX005, which suppresses C1q protein activity to block the classical complement cascade (Rajabally, 2022), has also been utilized as a therapy for autoimmune diseases, such as warm autoimmune hemolytic anemia and Guillain–Barré syndrome (Lansita et al., 2017). Nonsteroidal anti-inflammatory drugs, the most prevalent agents used to mitigate inflammation, act by inhibiting cyclooxygenase-2, which is responsible for reducing inflammatory mediators. Consequently, PrimeC, containing celecoxib as a nonsteroidal anti-inflammatory drug component, exhibits regulatory effects on inflammation (Salomon-Zimri et al., 2023). Alternative signal transduction pathways have been implicated in the inflammation response. For instance, Fasudil targets the Rho/ROCK signal pathway (Zhao et al., 2019), while MN-166 acts upon the cAMP-PKA pathway (Noor et al., 2007; Kikuchi et al., 2013), both effectively suppress inflammation through the blockade of signal transduction processes (Wang et al., 2022). FC-12738 is retro-iversion thymopentin that, similarly to thymopentin 5, interacts with toll-like receptors 2 to regulate neuroinflammation (Ellison, 2023). Notably, studies suggest that neuroinflammation may arise due to nucleoid TDP-43 loss and the subsequent increase in long interspersed nuclear element 1 activity (Liu et al., 2019; Bright et al., 2021). In this context, TPN-101 has been investigated as a potential therapeutic agent for C9orf72-ALS, as it inhibits long interspersed nuclear element 1 reverse transcriptase activity to prevent immune responses and protect cells. Moreover, research indicates MediCabilis CBD oil’s activation of cannabinoid 2 (CB2) could potentially alleviate neuroinflammation and oxidative stress (Alzforum, 2023). Despite the distinct targets of the eight drugs, they all act to reduce inflammation, which may be attributed to the comprehensive state of research on neuroinflammation in ALS pathogenesis.

*Anti-oxidative stress compounds*

Compounds that mitigate anti-oxidative stress represent the second highest proportion of ALS drugs, accounting for 25%. A total of six relevant clinical trials were collated, including one phase 2 (studying antioxidants, including vitamin E, NAc cysteine, L-cystine, nicotinamide and taurursodiol), four phase 3 (three investigating MT-1186 and one FAB122, which are different forms of edaravone), and one phase 2/3 (deferiprone). Among these six clinical trials, four are focused on the approved drug edaravone. Edaravone received FDA approval in 2017 (Gao et al., 2023); however, it is administered intravenously, and long-term intravenous administration may lead to secondary infections, allergies, infusion reactions, and other adverse reactions. MT-1186 (NCT04569084) and FAB122 (NCT05178810) are novel formations of edaravone that can be administrated orally. This route has the potential to reduce the side effects and costs of edaravone treatment. The four collected clinical trials aim to confirm the efficacy and safety of oral edaravone. In the phase 2 clinical trial NCT04244630, various anti-oxidative drugs (antioxidants) are being combined to enhance the efficacy and minimize side effects of the treatments. By preventing unstable non-transferrin-bound iron from entering plasma, deferiprone helps to mitigate ROS accumulation in cells (Devos et al., 2020; Merkel et al., 2024). A phase 2 clinical trial (NCT02164253) demonstrated that treatment with deferiprone resulted in a slower rate of decline in the Functional Rating Scale and body mass index of ALS patients; hence, deferiprone offers hope as a method of delaying disease progression.

*Metabolism modulators*

Imbalances in the homeostasis of energy metabolism is a risk factor for ALS and a significant contributor to disease progression (Burg and Van Den Bosch, 2023). Both trimetazidine dihydrochloride and CNMAu8 enhance electron transfer chain activity, which plays a pivotal role in adenosine triphosphate production (Kantor et al., 2000; Vucic and Kiernan, 2023). Particularly noteworthy is the catalytic activity resembling mitochondrial complex 1 exhibited by CNMAu8, an indispensable component of the electron transfer chain involving nicotinamide-adenine dinucleotide. Another common drug, ambroxol, which is mostly used for respiratory tract diseases, can modulate glucocerebrosidase activity, especially GBA2, to inhibit glucosylceramide synthesis (Bouscary et al., 2019). In preclinical trials, ambroxol delayed the symptom onset and promoted the life expectancy of SOD1 mice (Bouscary et al., 2020).

#### Non-disease-modifying therapy

The only medication for which data were gathered that has shown efficacy in relieving ALS symptoms is TJ-68, a traditional Chinese/Japanese herbal remedy containing *Paeonia Lactiflora* and *Glycyrrhiza Uralensis*. TJ-68 has been found to alleviate pain, spasms, and convulsions (Sakai et al., 2009). Although the mechanisms are not yet fully understood (Lee et al., 2018), TJ-68 is currently utilized for thrombosis, rheumatic arthritis, hyperuricemia, and even muscle cramps in Japan (Kumada, 1999). Given that muscle cramps are a prominent symptom of ALS, treating patients with TJ-68 may potentially improve their quality of life (Mitsumoto et al., 2023). Consequently, a phase 1/2 randomized placebo-controlled clinical trial (NCT04998305) is presently underway to assess the feasibility, safety, and efficacy of TJ-68.

#### Stem cell therapies

We identified three clinical trials related to stem cell research, one of which is currently in phase 1 (NCT05306457). Central nervous system 10-neural progenitor cells-glial cell derived neurotropic factor (CNS10-NPC-GDNF) refers to a specific type of programmed neural progenitor cell capable of producing GDNF, which acts as a growth factor for motor neurons. In the SOD1 rat model, these cells have the ability to differentiate into astrocytes within the spinal cord and exhibit a protective effect on motor neurons (Baloh et al., 2022).

Another trial, NCT03268603, currently in phase 2, is focused on adipose-derived mesenchymal stromal cells, stem cells derived from adipose tissue that offer advantages over bone marrow mesenchymal stem cells due to their abundance and accessibility. Adipose-derived mesenchymal stromal cells possess regenerative, paracrine, and immunomodulatory properties, making them valuable for wound healing as well as the treatment of cardiovascular and rheumatic immune disorders (Czerwiec et al., 2023). Studies have demonstrated their potential to differentiate into neurons and secrete nerve growth factors such as GDNF (Laloze et al., 2021), suggesting they could have therapeutic relevance in ALS. Moreover, the functional potential of adipose stem cells can be enhanced through accessory attachment. For instance, adipose stem cell exosomes have demonstrated neuroprotective properties, potentially attributed to the exosomal proteins’ ability to facilitate cell adhesion and regulate apoptosis (Bonafede et al., 2019).

The final candidate, currently in a phase 3 clinical trial, is lenzumestrocel, a stromal stem cell therapy derived from bone marrow that has been approved by the Korean Ministry of Food and Drug Safety. Similar to adipose-derived mesenchymal stromal cells, this therapeutic agent possesses both differentiation capacity and secretory function, enabling it to produce neurotrophic factors. Lenzumestrocel has demonstrated the ability to modulate inflammation in ALS (Nam et al., 2023).

### Preclinical drugs

While this article’s primary focus is on an analysis of drugs in clinical development, it is important to note that investigating preclinical drugs can also yield valuable insights into drug development trends, providing possible experimental development directions. During the trial periods, four preclinical drugs demonstrated remarkable efficacy in slowing disease progression. A specific study conducted on animal models revealed that exosomes derived from human MSC544, a continuously proliferating human mesenchymal stem cell line containing miRNAs responsible for regulating antioxidant and anti-apoptotic pathways, exhibited protective effects on SOD1^G93A^ ALS primary motor neurons. Furthermore, as a cell-free product, it displayed safety and processing advantages over cell therapy during clinical trials (Gschwendtberger et al., 2023). Another study demonstrated the protective effects of honokiol in motor neurons; honokiol protected against mutant SOD1^G93A^ toxicity, ameliorating mitochondrial dysfunction through its inhibition of oxidative stress, promotion of mitochondrial biogenesis, and maintenance of the mitochondrial fusion-fission balance by acting via the NRF2/GSH signaling pathway, which underscores the potential advantages of multi-target therapy for ALS (Zhou et al., 2023). Additionally, ATH-1105 small molecules act as regulators of the neural nutrient HGF system and can be orally delivered throughout the body. They have demonstrated the ability to safeguard various cell culture systems from glutamic-acid-induced toxicity and related pathological changes in a Prp-TDP43^A315T^ transgenic mouse model, while also exhibiting potent anti-inflammatory and neuroprotective effects (Berthiaume et al., 2024). Montelukast, a safe drug with current market availability, has demonstrated therapeutic potential in ALS by exerting anti-inflammatory effects and fostering a conducive glial microenvironment for central nerve repair. This intervention significantly extended the survival of SOD1^G93A^ mice and improved their motor function (Raffaele et al., 2024).

## Other Potential Therapies

### Gene therapy

#### Antisense oligonucleotides

Studies have demonstrated that the toxic GOF effect of mutated genes is frequently the pathological mechanism underlying numerous monogenic mutated ALS (Kim et al., 2020). For these cases, therapeutic strategies that directly modify the causative gene and thereby diminish or neutralize the toxicity hold considerable promise (Boros et al., 2022). An ASO is a synthetic sequence of oligonucleotides that selectively binds to mRNA and modifies protein synthesis through mechanisms such as mRNA degradation, micro-RNA inhibition, exonic slicing modulation, and ribonuclease H activation (Crooke et al., 2018). ASOs regulate gene expressions without changing DNA, achieving a steric block that promotes RNA cleavage and degradation. Nevertheless, they still demonstrate certain toxicities that can activate the immune and complement systems while also influencing coagulation processes (Gheibi-Hayat and Jamialahmadi, 2021). Despite challenges related to the immune stimulation properties and delivery to the central nervous system of some current ASO therapies, and given the potent therapeutic potential of ASOs for numerous prevalent diseases, addressing improvements to their efficacy and mitigating their side effects are worthwhile challenges. ASOs can be chemically modified, through cholesterol conjugation for example, and new delivery methods are being explored, such as lipid nanoparticles (Kulkarni et al., 2018; Nagata et al., 2021).

*Superoxide dismutase 1*

As previously mentioned, the approved drug tofersen is an ASO designed with the aim of reducing SOD1 protein levels for the treatment of SOD1-ALS patients, and initially brought hope for a successful gene therapy (Miller Timothy et al., 2022). However, the delay between clinical and biomarker responses in clinical trials and the revelation that the long-term suppression of the normal SOD1 allele may be harmful remind us of the need for the long-term follow-up of patients (Van Daele et al., 2024).

*Chromosome 9 open reading frame 72*

Apart from the current SOD1 studies, ongoing research is focused on developing ASOs targeting genes associated with C9orf72 (Jiang et al., 2016; Becker et al., 2017). However, a recent study (van den Berg et al., 2024) reported that BIIBo78, an ASO for C9orf72-associated ALS in phase 1 trial, showed no ability to reduce neurofilament levels and no benefit to clinical outcomes relative to the placebo cohort, which has revealed the limitations of the gene-silencing method and the necessity of exploring multi-mechanism combination therapies. Currently, the reasons for the disappointing outcomes are under discussion, such as the possibility that ASOs might influence the expression level of wild-type C9orf72. Although these ASOs have been designed to avoid affecting the normal expression levels of C9orf72 protein, the effect in patients seem to be consistent with the negative effects observed in mice (Zhu et al., 2020), such that the LOF hypothesis takes precedence. Alternatively, the antisense repeat mRNA might be more significant than anticipated, and the breakdown of sense RNA could impact its efficacy. Although numerous results remain unexplained, these discussions will play a crucial role in subsequent reflective validation tests.

*Fused in sarcoma*

A small-sample clinical trial of the drug ION363 is currently in its third phase. The study was initiated in response to the conclusion that FUS-mediated toxicity is not a consequence of LOF, but rather a toxic GOF associated with the pathogenic mutation, and that the degree of cytoplasmic mislocalization does not correlate with the *in vivo* toxicity of the mutant FUS. It is expected to be completed by 2028 (Korobeynikov et al., 2022). Furthermore, three patients with FUS-ALS were granted approval by the FDA to undergo treatment with such drugs, while another eight patients with ALS are under investigation (Amado and Davidson, 2021; Cappella et al., 2021).

*TAR DNA-binding protein 43*

TDP-43 is a highly conserved nuclear RNA/DNA-binding protein implicated in the regulation of RNA processing (Jo et al., 2020). Under pathological conditions, TDP-43 can undergo cleavage, hyperphosphorylation, and ubiquitination (Gasset-Rosa et al., 2019). These post-translational modifications result in the pathological cytoplasmic accumulation and aggregation of TDP-43, which can directly induce the nucleocytoplasmic transport and disruption of nuclear pore complexes, further promoting the mislocalization and accumulation of TDP-43 in the cytoplasm and ultimately causing neuronal dysfunction and toxicity (Neumann et al., 2006). This mechanism makes it tempting to compare TDP-43 with therapies that directly degrade abnormally aggregated TDP-43 proteins; nevertheless, there is a prevailing consensus that reducing the overall level of TDP-43 is deleterious (Igaz et al., 2011; Polymenidou et al., 2011; Tsao et al., 2012). Logically, it is not feasible for an ASO therapy to directly target the *TARDBP* gene, but rather it should effect key pathogenic mRNA. The *STMN2* gene represents one of these opportunities, as it encodes the stathmin-2 protein, which is crucial for the spatial stability and transport function of neurons (Krus et al., 2022; Baughn et al., 2023). A previous study demonstrated that the depletion of TDP-43 leads to the decreased binding of TDP-43 to STMN2 pre-mRNA and the concurrent production of nonfunctional stathmin-2 mRNA, leading to reduced levels of functional stathmin-2, which seemingly contributes to the pathogenesis of ALS (Elden et al., 2010; Pottier et al., 2019). Another candidate is UNC13A, which influences the release of synaptic vesicles and is anticipated to lead to premature codon termination, potentially triggering nonsense-mediated decay (Brown et al., 2022). It has been proclaimed that an ASO capable of correcting the cryptic exon insertions resulting from mis-splicing has been identified that can thereby restore the production of UNC13A protein (NCT05633459). This advancement is anticipated to be beneficial for the majority of patients presenting with the pathological phenomenon of abnormal TDP-43 protein aggregation.

*ATXN2*

Another approach lies in down-regulating genetic risk factors for ALS, such as ATXN2, whose mutations give rise to progressive familial spinocerebellar ataxia type 2, a conditions linked to a significantly elevated risk of ALS (Elden et al., 2010). In a preclinical study, the treatment of TDP-43 mouse models with ASO led to improved motor performance and increased survival. Hence, a phase 1/2 trial was initiated to assess the safety and efficacy of BIIB105, a drug designed to reduce ATXN2 protein expression (NCT04494256). However, by August 2024, the trial had failed to improve the patients’ condition. While BIIB105 effectively decreased ATXN2 protein levels, it failed to reduce the amount of neurofilaments. Regrettably, the development of this experimental drug for the treatment of ALS has thus been halted.

#### Small-interfering RNA

SiRNA represents a gene therapeutic approach that shares similarities with ASOs, as it exerts its influence on gene expression by selectively targeting and cleaving specific RNA molecules, leading to their silencing. However, due to the inherent instability and lack of specificity of the approach, further investigations are warranted to enhance its applicability (Hannon and Rossi, 2004). A preclinical trial of siRNA-ACO (Accessory Oligonucleotide) conjugate RAG-17 may throw light on the potential of siRNA therapy by showing its great stability and biodistribution activity, ability to inhibit SOD1 gene expression, and tendency to extend the SOD1 mouse lifespan (Duan et al., 2023).

#### Vectors

To exert their functionality within the central nervous system, ASO and siRNA methods necessitate the use of carriers that facilitate their traversal across the blood–brain barrier and cell membrane, ensuring protection against degradation while maintaining their stable expression in neurons. Consequently, we present two distinct categories of carriers: non-viral vectors and viral vectors.

*Non-viral vectors*

Non-viral vectors encompass a diverse range of types, with nanotechnology-based vectors being particularly popular. Notably, the DMT drug CNMAu8 is also classified as a gold nanocrystal. The prevalence of nanotechnology can be attributed to several factors. First, the latest generation of nano vectors are designed to evade immune responses and ensure the stability of therapeutic agents (Fadeel, 2019). Second, surface modifications enable nanoparticles to effectively traverse the blood-brain barrier and selectively bind to target sites within neurons, therefore enhancing the agent concentration. Third, nanomaterials exhibit a high capacity for therapeutic agents and demonstrate remarkable stability (Ediriweera et al., 2021). To date, various nanomaterials have been employed in ALS research, including liposomes, solid inorganic nanoparticles, carbon nanotubes, polymeric nanoparticles, exosomes, and solid lipid nanoparticles (Yetisgin et al., 2020).

*Viral vectors*

Viral vectors represent a promising avenue for gene therapy, particularly in the context of rare diseases, owing to their ability to traverse the blood-brain barrier, inherent stability, and robust cloning capabilities (Cappella et al., 2019). In ALS, lentivirus (LV) and adeno-associated virus (AAV) vectors have emerged as prominent agent carriers. These vectors possess simplified genomes, with the exclusion of genes associated with oncogenesis and apoptosis to minimize potential harm to humans (Escors and Breckpot, 2010; Samulski and Muzyczka, 2014). Early investigations have also demonstrated the favorable effects of viral vectors on ALS survival (Raoul et al., 2005; Foust et al., 2013; Borel et al., 2018). Compared to LV, AAV exhibits a tissue-specific tropism, with serotype 9 and rh10 particularly targeting neurons, and is the most extensively utilized for ALS (Cappella et al., 2019).

### Immunotherapy

Early investigations have substantiated the involvement of immune cells, the complementary system, and cytokines in the pathogenesis and progression of ALS (Yang et al., 2021). In this context, our previous study demonstrated that the FDA-approved drugs fostamatinib and amlexanox have the potential to be promising avenues for ALS treatment. These act by mitigating neuroinflammation in ALS cell models with overly active cGAS/STING signaling through the inhibition of TBK1 phosphorylation (Duan et al., 2024). Moreover, the administration of a C5a antagonist has been shown to extend the survival of SOD1 mutant mice (Woodruff et al., 2014). Additionally, targeting the C-terminal of TDP-43 with a monoclonal antibody can effectively eliminate misfolded TDP-43 aggregates and mitigate neurotoxicity (Afroz et al., 2023). Notably, an active immunotherapy demonstrated efficacy in a mouse model harboring C9orf72 mutation; treatment with the poly-glycine-alanine vaccine resulted in reduced levels of misfolded TDP-43, alleviated neuroinflammation, and attenuated neuronal damage (Zhou et al., 2020). Elevated levels of natural killer cells and inflammatory biomarkers (IL-2, -5, and -8) result in neurotoxic effects, whereas an increase in regulatory T cells is associated with improved survival among patients with ALS (Maharaj et al., 2023). The immune-mediated mechanisms underlying the pathogenesis of ALS are still under investigation; therefore, targeted immunotherapies remain a crucial aspect of the pursuit of ALS treatments.

## Prospects

In this article, we have comprehensively documented the recent advancements made in therapeutic research for ALS. The majority of the drugs exhibit well-defined mechanisms of action; however, a few compounds, such as TJ-68, possesses unclear molecular pathways, and investigations into the specific mechanisms of MediCabilis CBD Oil is undergoing. Nonetheless, it is imperative to emphasize that elucidating and enhancing our understanding of the pathological mechanisms underlying ALS serve as the fundamental basis for innovative drug research. Therefore, continued efforts should be directed towards exploring novel pathological pathways. To date, the FDA has approved five drugs that delay the progression of ALS. We also elaborated on three other clinical trials, all of which are interested in oral edaravone (MT-1186). These have explored novel perspectives on drug development, prompting explorations of alternative administration approaches and dosage forms that might potentially provide expedited benefits to patients. Furthermore, within the realm of DMT drugs, our collected data predominantly highlighted the role of anti-inflammatory agents targeting diverse signaling cascades. To enhance their efficacy and minimize side effects, a potential strategy is to combine drugs that target different pathways or targets such as antioxidants and AMX0035. Regarding DMT drugs, approximately half exhibit relevance to TDP-43, with five directly targeting this protein. Given that abnormal TDP-43 is present in over 90% of ALS patients, therapeutics capable of clearing pathological TDP-43 may hold promise as universal treatments for diverse forms of the condition. However, considering the limited sample size in each trail, the data sources and outcomes remain biased. Some drugs have demonstrated considerable potential in preclinical trials, yet the outcomes of phase 2 and 3 trials have been unsatisfactory. This has led to a tumultuous journey for the emergence of orphan drugs for MNDs, and the failure to update the experimental results has also complicated analyses of drug pipelines. Furthermore, if an analysis is solely reliant on website data, then merely the predicted time is used, as the precise completion time and specific experimental outcomes are lacking, which cannot yield an accurate study of drug performance. We can merely catch a glimpse of the recent progress, drawing clues from one trial after another and relying on the altruism of the research groups. Gene therapy has emerged as a prominent area of investigation among other treatment modalities. However, current research indicates that most cases of ALS are sporadic, with only a subset of patients exhibiting genetic alterations. Owing to the complex etiology and significant heterogeneity of ALS, the existing drugs and approaches available in the market are not always applicable. Consequently, efficiently implementing precise and individualized treatments for patients with diverse types of mutation remains an ongoing challenge. Identifying the key mechanisms underlying common pathological pathways is of paramount importance, and a comprehensive evaluation of the symptoms, course, and genetics of each patient is indispensable.

## Data Availability

*Not applicable*.
